# Blantyre-Oslo Neurosurgery Program

**DOI:** 10.3402/gha.v9.32016

**Published:** 2016-06-03

**Authors:** Signe Marie Bandlien, Liz Palm

**Affiliations:** 1 Fredskorpset Norway, signe@fredskorpset.no; 2 Fredskorpset Norway, liz.palm@fredskorpset.no

**Figure:**
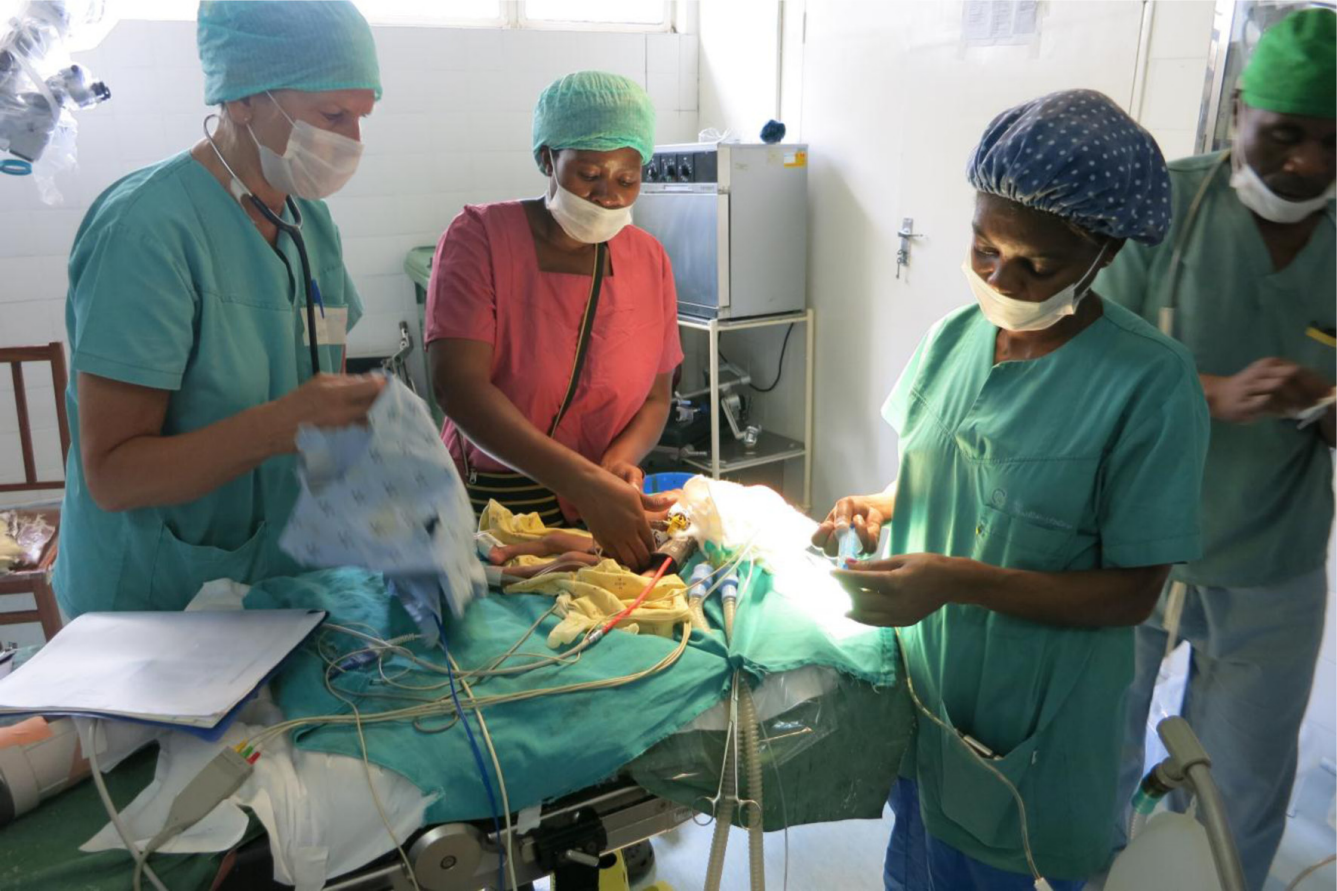
Photographer: Signe Marie Bandlien, FK Norway.

An institutional health partnership collaborating to improve the quality of service delivery and education within the fields of nursing, anesthetics, and neurosurgery in Norway and Malawi.

The project aims to establish a competent neurosurgical service in Malawi and train Norwegian health personnel in global health challenges. An additional project aim is to improve the Norwegian health system for immigrants. Fredskorpset Norway's (FK Norway) exchange program for professionals is the methodology through which capacity building is accomplished, through exchange of staff between the neurosurgical departments at Oslo University Hospital in Oslo and Queen Elizabeth Central Hospital in Blantyre (QECH).

The project began 3 years ago, and now the unit is up and running in Malawi. The picture shows the Norwegian–Malawian team at work in the operating theatre at QECH. It shows the preparations for surgery on a premature baby on an early Monday morning in March 2015. The project is now in a phase which will expand to include the pediatric surgery units in both hospitals and continue their training program for ward nurses, ICU nurses, clinical officers, and surgeons for the Norwegian and Malawian clinical staff.

The project is financially supported by the Norwegian government through FK Norway and Oslo University Hospital. It is supported by both hospitals’ leadership through facilitating staff to go on exchange, providing dedicated resource personnel and senior staff guidance, and encouraging the peer networks both within Malawi and Norway, and between the two countries, to grow. The program is in line with both nations’ national health authorities’ strategies.

*Signe Marie Bandlien* Fredskorpset Norway signe@fredskorpset.no
*Liz Palm* Fredskorpset Norway liz.palm@fredskorpset.no

